# The impact of AMniotic FluId on the development and microBIal colonization of the prEterm intestinal tract (AMFIBIE): study protocol for a multicenter prospective cohort study

**DOI:** 10.3389/fped.2025.1721519

**Published:** 2026-01-12

**Authors:** Rimke R. de Kroon, Sofie van Weelden, Loes Monen, Petra Bakker, Eva Pajkrt, Eduard A. Struys, Andries E. Budding, Tim de Meij, Hendrik J. Niemarkt, Mirjam M. van Weissenbruch

**Affiliations:** 1 Amsterdam Reproduction & Development, Amsterdam UMC, Amsterdam, Netherlands; 2 Amsterdam Gastroenterology Endocrinology Metabolism Amsterdam, Amsterdam UMC, Amsterdam, Netherlands; 3 Pediatric Gastroenterology, Emma Children’s Hospital, Amsterdam UMC – Location AMC, Amsterdam, Netherlands; 4 Gynaecology & Obstetrics, Máxima MC, Veldhoven, Netherlands; 5 Gynaecology & Obstetrics, Amsterdam UMC – Location AMC, Amsterdam, Netherlands; 6 Department of Laboratory Medicine, Amsterdam UMC – Location AMC, Amsterdam, Netherlands; 7 inBiome, Amsterdam, Netherlands; 8 Neonatal Intensive Care Unit, Máxima MC, Veldhoven, Netherlands; 9 Neonatal Intensive Care Unit, Emma Children’s Hospital, Amsterdam UMC – Location AMC, Amsterdam, Netherlands

**Keywords:** amniotic fluid, chorioamnionitis, metabolomics, microbial profiling, necrotizing enterocolitis, neonatal gut colonization, sepsis

## Abstract

**Introduction:**

Prematurity is associated with intestinal immaturity and gut microbiota alterations, both of which are linked to necrotizing enterocolitis (NEC) and sepsis. An important yet understudied contributor in the development of the gastrointestinal (GI) tract is amniotic fluid (AF). The aim of this study is to assess the composition of AF collected during extremely preterm birth. Secondary objectives are to identify AF profiles of pregnancies complicated with chorioamnionitis and/or fetal growth restriction, assess key AF components across gestation, correlate AF profiles with neonatal outcomes (e.g., NEC and sepsis), and explore associations with neonatal gut microbiota.

**Methods and analysis:**

In this multicenter prospective cohort study, AF (∼5 mL) will be collected from obstetric patients delivering their infants extremely preterm [gestational age (GA) 24 + 0/7–27 + 6/7 weeks, *n* = 125]. AF can be collected safely and non-invasively during vaginal delivery or cesarean section. AF samples will also be collected from a reference group (GA <23 + 6/7 and 28 + 0/7–40 + 6/7 weeks, *n* = 150). Characterization of AF will include microbial and metabolic profiling. By advancing our understanding of the role of AF in the GI development and neonatal disease, this study may contribute to the early identification of infants at risk to develop NEC and/or sepsis. Ultimately these findings may facilitate early targeted microbiota-based interventions to prevent disease progression and improve outcomes. While the current study provides clinical opportunities, several methodological challenges inherent to collecting AF at birth must be acknowledged, including variability in sampling method, maternal blood interference, and the risk of microbial contamination.

**Ethics and disseminations:**

Ethical approval was received by the METC of the Máxima Medical Center in Veldhoven, the Netherlands (W24.042). The study was registered in a public clinical trial registry (NCT07152106). Study findings will be disseminated through peer-reviewed journal articles as well as national and international conference presentations.

**Clinical Trial Registration:**

https://clinicaltrials.gov/study/NCT07152106, identifier NCT07152106.

## 1 Introduction

### 1.1 Background

Compared to term infants, infants born extremely preterm [gestational age (GA) <28 weeks] often display intestinal immaturity and gut microbiota alterations, which play a prominent role in the development of necrotizing enterocolitis (NEC) and sepsis (1–4). An important yet understudied contributor to the development of the fetal intestines is amniotic fluid (AF) (5). Reduced exposure to trophic factors in AF, as is the case for preterm infants, may compromise healthy intestinal development and colonization. However, knowledge is lacking on the composition of AF of extremely preterm infants and its effect on the gut microbiota. We hypothesize that elucidating the composition of AF, its effects on the neonatal intestines, and its role in the initiation of gut microbiota colonization will increase our understanding of the etiology of NEC and sepsis, facilitate the identification of perinatal biomarkers, and open a window of opportunity for preventive strategies.

AF is a dynamic biological fluid, which plays a pivotal role in fetal development, ranging from functioning as a protective fluid against physical trauma, to contributing to fetal nutrition and aiding in the protection against fetal infection (6). The composition of AF reflects the health of mother and fetus, highlighting the value of AF as a tool in the diagnosis of a wide spectrum of clinical conditions (7). While AF is most well-studied for its role in fetal lung development, AF is also a contributor to the development of the fetal gastrointestinal (GI) tract as the intestines are exposed to various bioactive components in AF through swallowing, starting around the 12th week of gestation. Previous studies have demonstrated that various trophic factors are present in AF that impact the fetal intestinal epithelium, including epidermal growth factor, hepatocyte growth factor, insulin-like growth factor-1, erythropoietin, and various interleukins (6, 8). Other components in AF, including amino acids, lipids and fatty acids, cytokines, and potentially even microorganisms in the case of microbial invasion of the amniotic cavity [e.g., as a result of preterm premature rupture of membranes (PPROM)], may also impact the development of the fetal gut and subsequent intestinal maturation in early life. However, the functions and significance of these individual components in AF impacting the intestinal epithelium *in utero* remain incompletely understood.

Previous studies on the characterization of AF through metabolomic, proteomic, and transcriptomic analyses have demonstrated that the content of AF and concentration of bioactive components gradually change over the course of pregnancy (9, 10). However, knowledge remains especially limited regarding the potentially critical changes that occur in the AF composition between the 24th and 28th week of gestation and the impact of these changes on the fetal GI tract. This gap in knowledge is particularly concerning, as infants born during this period are most susceptible to develop GI-related diseases, including NEC and sepsis. Hence, we aim to address this knowledge gap through characterization of AF of extremely preterm infants using various biomedical techniques. To do so, we initiated the AMFIBIE study, acronym for *The impact of AMniotic FluId on the development and microBIal colonization of the prEterm intestinal tract*, in October 2024. The AMFIBIE study is a multicenter prospective cohort study collecting AF from mothers delivering their infants extremely preterm (GA 24 + 0/7–27 + 6/7 weeks), during vaginal delivery or cesarean section (CS). Two subgroups are of particular interest due to their association with neonatal morbidity and mortality: preterm infants exposed to chorioamnionitis (CAM), and preterm infants with fetal growth restriction (FGR). Notably, AF will also be collected from a reference group (GA ≤23 + 6/7 weeks and GA 28 + 0/7–40 + 6/7 weeks).

The first group of interest consists of preterm infants exposed to CAM, which is one of the major causes of preterm birth (11). Fetal exposure to CAM is associated with neonatal morbidity, including NEC and sepsis (12). *In vivo* studies in animal models have shown that *in utero* exposure of the fetal GI tract to pathogens and inflammatory mediators in AF is associated with altered development of the intestinal barrier (13). Exposure to CAM may also result in increased susceptibility to intestinal injury (14), providing a possible explanation for the correlation found between gut-associated diseases and CAM. However, the role of fetal intestinal exposure to AF and its impact on gut neonatal gut colonization remains largely unexplored. Identification of CAM-exposed infants is difficult due to non-specific clinical presentation and subclinical manifestation, even with intact fetal membranes. There are two commonly used definitions for CAM, *histopathologic* CAM and *clinical* CAM. *Histopathologic* CAM is confirmed by placenta histopathology. However, the report may take up to 6 weeks (15). Notably, placental signs of CAM are common in the preterm population, while *clinical* CAM, characterized by maternal fever, leukocytosis, tachycardia, uterine tenderness, and/or preterm rupture of membranes, is less frequent (16). Various pathogens, such as *Ureaplasma* species, *Gardnerella vaginales*, *Bacteroides*, Group B *streptococcus*, and *Escherichia coli*, have been isolated from AF, as demonstrated in a study on patients undergoing amniocentesis in the second trimester (17). However, traditional culturing is challenging and molecular techniques yield more promising results (18). Moreover, few studies have focused on metabolic profiling (7). Data on the composition of AF of specifically extremely preterm infants is limited, largely due to challenges in the sample collection during preterm delivery.

The second group of interest consists of growth-restricted infants. FGR is defined as an inability of the fetus to grow to its expected biological potential *in utero* (19). FGR is associated with preterm birth and various neonatal morbidities, including NEC and sepsis (20). In response to lack of oxygen and nutrients, fetal cardiovascular adaptation will take place to ensure brain protection and altered development of the fetal GI tract (21). Animal studies have shown that FGR impacts the small intestines and may result in increased susceptibility to GI disorders (22). Alterations in gut microbiota have been demonstrated in growth-restricted infants (23). Previous studies on AF have shown that various metabolites demonstrate a significant change in abundance associated with the second-to-third-trimester transition. These changes may correlate with the stabilization of fetal growth during this period (10), raising our interest in AF composition in growth-restricted infants and its impact on the development of the preterm intestines.

Being born extremely preterm lays a pivotal foundation for short-term and long-term consequences. We argue that elucidating the role of inflammation and microbiota in the perinatal period will not only increase our understanding of disease pathogenesis, but may also aid in the identification of infants at increased risk for NEC and sepsis. Early risk stratification may eventually result in early targeted intervention, such as selective antibiotic therapy, tailored nutrition, or other preventative strategies, and improve neonatal clinical outcomes. Hence, we present the study protocol for an exploratory, hypothesis-generating study aimed at identifying associations between AF exposure, preterm birth, and early gut microbial colonization. We propose that characterizing AF obtained at or just before preterm delivery may offer valuable (patho)physiological insights. The current study offers an opportunity to investigate the microbial and metabolic profiles of AF collected at birth and to explore its potential clinical value in early risk stratification. However, several methodological challenges inherent to collecting AF at birth must be acknowledged, including variability in sampling method, maternal blood interference, and the risk of microbial contamination. In light of the longstanding debate regarding the existence of a fetal and/or prenatal intrauterine microbiome (24), particular transparency is required in addressing potential microbial contamination of these low-biomass samples, subsequent downstream analyses, and interpretation of findings. These opportunities and challenges, and the strategies we apply to address them, are outlined in detail below.

### 1.2 Objectives

The primary objective of the current study is to characterize the microbial and metabolic composition of AF collected during extremely preterm delivery (GA 24 + 0/7–27 + 6/7 weeks).

*Secondary objectives include*:

To compare the AF profiles of extremely preterm infants with and without CAM.
To compare the AF profiles of growth-restricted infants to non-growth restricted infants.
To assess the course of key AF components across GAs.
To correlate the AF profiles of extremely preterm infants, identified in objective 1 and 2, to the composition of neonatal gut microbiota in the first month of life.
To correlate AF profiles to neonatal morbidity, including NEC and sepsis in the first month of life, and identify potential AF-based biomarkers.

## 2 Methods and analysis

### 2.1 Design and setting

**Figure 1:**
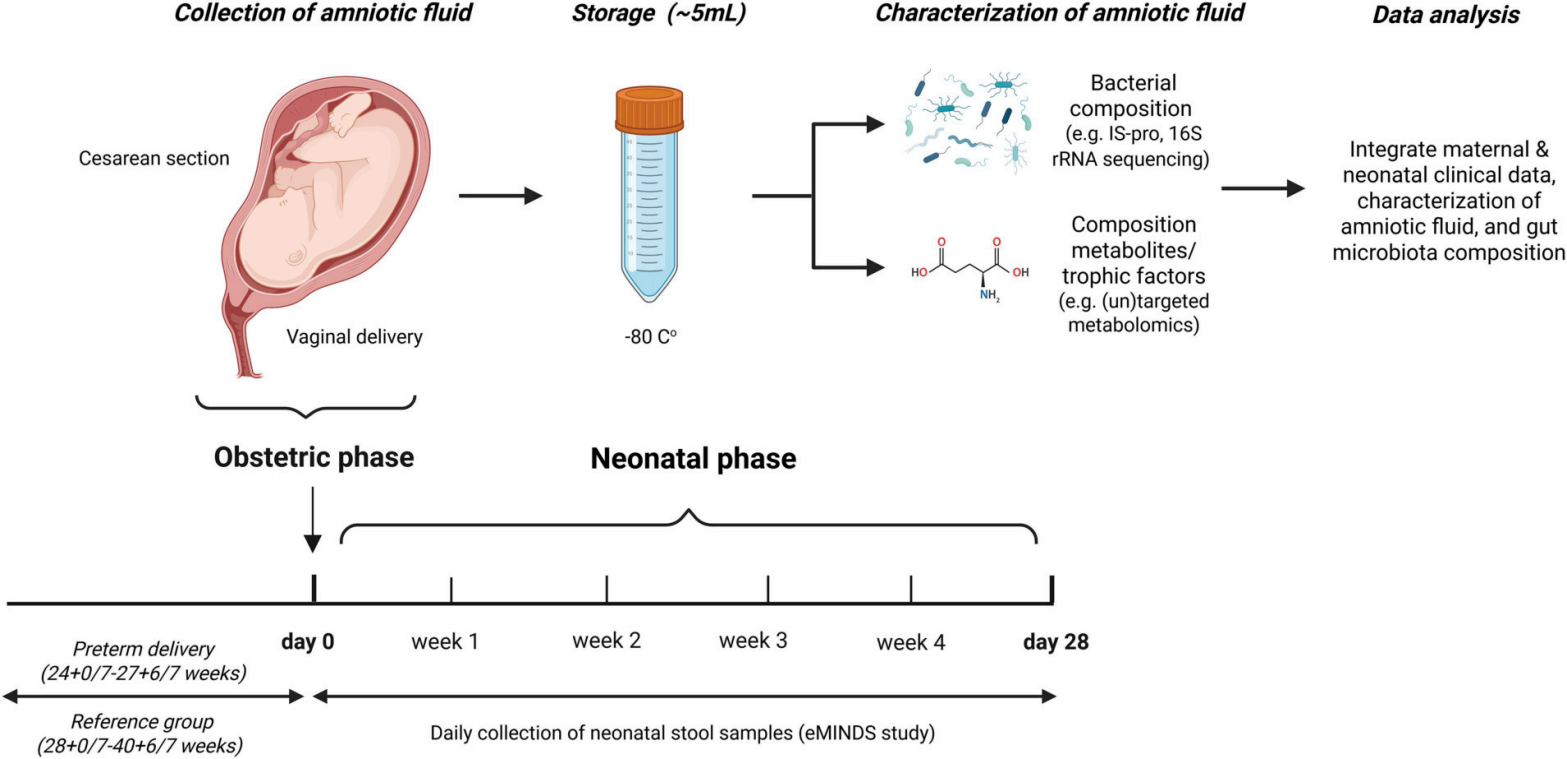
Schematic overview of the AMFIBIE study. Created with Biorender.com.

Obstetric patients are recruited from two perinatological tertiary centers in the Netherlands, the Máxima Medical Center and the Amsterdam UMC. The study is conducted in two phases for each mother-infant pair in the study group (GA 24 + 0/7–27 + 6/7 weeks, *n* = 125) (Table 1, Figure 1). In phase 1 (*obstetric phase*), AF (∼5 mL) will be collected during delivery. In phase 2 (*neonatal phase*), neonatal fecal samples will be collected. For phase 2, we will collaborate with the eMINDS study [Metc2014.386 (A2020.190)], acronym for *Enteral Microbiota and metabolomics in Infants for prediction of NEC, neuromotor Development and late-onset Sepsis*. The aim of this prospective cohort study is the development of predictive fecal biomarkers for NEC and sepsis. In addition to clinical data collection, fecal samples are collected daily in the first month of life for all live-born infants with GA 24 + 0/7–27 + 6/7 weeks. The recruitment for this study is ongoing at time of the AMFIBIE study.

**Table 1:** Overview mother-infant pairs in the study population.

Study group: gestational age 24 + 0/7–27 + 6/7 weeks (extremely preterm pregnancies)		
Variable	Obstetric phase	Neonatal phase
Number	125	125^a^
Sample type	Amniotic fluid sample collected during delivery	Meconium as well as fecal sample obtained on day 7, day 14, day 21, and day 28
Clinical data	Clinical and demographic data	Clinical and demographic data

^a^ In the unlikely case that no informed consent is provided by parents and/or guardians for participation in the eMINDS study and therefore fecal samples cannot be collected, obstetric patients will not be excluded from the AMFIBIE study.

### 2.2 Study population

To be eligible to participate (Table 2), the obstetric patient must be ≥16 years of age, AF has to be successfully collected during delivery, and informed consent must be obtained. Exclusion criteria are pregnancies complicated with major fetal congenital and/or chromosomal comorbidities, as well as insufficient proficiency of the Dutch or English language. Infants included in the study group must be born between 24 + 0/7 and 27 + 6/7 weeks (*n* = 125). AF samples are also collected from a reference group (Table 3), including early midtrimester (GA ≤23 + 6/7 weeks), very and moderate to late preterm (GA 28 + 0/7–36 + 6/7 weeks), and full-term pregnancies (GA 37 + 0/7–40 + 6/7 weeks) (*n* = 150).

**Table 2:** In- and exclusion criteria for the study group and reference group.

Inclusion criteria	Exclusion criteria
Maternal age ≥16 years	Pregnancies complicated by fetal congenital and/or chromosomal abnormalities.
Written informed consent	
Successful collection of amniotic fluid	Insufficient proficiency of Dutch or English language

**Table 3:** Overview of the collection of amniotic fluid for the study and reference group.

Subgroups	Gestational age (weeks)	Study group	Reference group^a^	Methods
Early mid-trimester	≤23 + 6/7	n.a.	60	Amniocentesis (clinical indication)
Early preterm	24 + 0/7–27 + 6/7	125	n.a.	Vaginal delivery, CS
Very, moderate and late preterm	28 + 0/7–36 + 6/7	n.a.	90	Vaginal delivery, CS
Full-term	37 + 0/7–40 + 6/7	n.a.		Vaginal delivery, CS
Total		125	150	

CS, cesarean section.

^a^ For the reference group, we aim to include approximately 6 infants per GA week in the very, moderate and late preterm as well as full-term reference group, resulting in approximately 90 infants.

### 2.3 Sample size estimation

A formal power analysis cannot be performed as there is currently insufficient literature available. The incidence of CAM varies from ∼25% to 40% and of FGR from ∼20% to 30% in preterm birth (25, 26). Due to the exploratory nature of this study, we aim to include ∼30 CAM-exposed and ∼25 growth-restricted infants in the extremely preterm group, resulting in an expected sample size of 125. Additionally, we expect to include 19 infants with sepsis (expected incidence 15%) and 9 with NEC (expected incidence 7%) (28, 29). Notably, the prevalence of vaginal vs. CS deliveries may differ between subgroups. For instance, infants affected by FGR are more frequently born through CS (27). As this is one of the first large-scale study collecting AF during extremely preterm deliveries, it remains to be determined whether the planned sample size will be sufficient for all planned subgroup analyses. Additionally, the incidence of CAM and FGR, on which the sample size calculation was based, may be lower than previously reported in literature. Assuming lower-end incidence rates of CAM (∼20%) and FGR (∼15%), in a cohort of 125 infants we would expect around 25 CAM-exposed infants and 19 growth-restricted infants. Reduced inclusion of these groups of interest may impact specific subgroup analyses. If our analyses only provide preliminary signals rather than robust conclusions, these findings may guide hypothesis generation and the design of future larger-scale validation studies.

### 2.4 Informed consent procedure

The obstetric patients will be preferably recruited by a member of the research team. As preterm delivery is often unexpected and requires acute handling with extensive counseling on various topics, it may not always be possible to immediately obtain written informed consent prior to the collection of the AF sample. Due to the non-invasive and safe nature of the study, if it is not possible to obtain full written informed consent prior to collection, AF may be collected during delivery by the obstetric caregiver. However, following AF collection, written informed consent must be obtained from the participant prior data collection or sample analysis.

### 2.5 Sample collection

AF, typically discarded during vaginal or cesarean delivery, can be safely and non-invasively collected without additional burden to the patient, for example during membrane rupture or routine cervical examination. Exclusively sterile materials are used for the collection of AF. For instance, when the membranes rupture, AF can be collected directly into a sterile collection cup or into a sterile, disposable kidney tray by the obstetric health care provider. As transient exposure to a larger open surface, as occurs when using a single-use kidney tray, may increase the risk of contamination, direct collection into a single-use collection cup is preferred. If a kidney tray must be used as an intermediate container (for example, if positional factors prevent direct collection into the cup), exposure time should be minimized, and trays should be handled under strict aseptic conditions. Additionally, when performing routine cervical examinations, any AF that is present on the palm of the sterile gloved hand can also be carefully transferred into the collection cup. During vaginal delivery there is an increased risk of contamination by the maternal urogenital microbiome. The mode of collection and risk of contamination will be taken into account when interpreting the findings.

During CS, AF can be obtained through the intact membranes (the preferred method). A sterile canula is carefully inserted through the amniotic membranes, following the hysterotomy but before amniotomy, after assessment of the position of the neonate. AF is then aspirated and transferred to the collection cup. The collection of AF through intact amniotic membranes has been described in various publications, none of which have reported complications (such as damaging the skin or eyes of the neonate or hampering the progress of the CS) (30–36). If the obstetric doctor does not wish to drain AF through the intact membranes, collection of AF may instead be performed directly after rupturing of the amniotic membranes, either directly into the sterile collection cup or using a 10 mL sterile syringe for transfer. A potential theoretical risk includes delay of delivery due to collection. However, these risks are deemed minimal based on available literature. The obstetrician is responsible for making a well-informed decision on whether it is feasible to safely collect AF.

If there is a clinical indication to conduct an amniocentesis (≤23 + 6/7 weeks), a few milliliter of additional AF can be collected for study purposes. In the case of left-over AF not required for clinical diagnostics, the residual fluid can also be used as reference material. Amniocentesis will be performed conform the local guidelines, posing no additional stress or risks for the participant of the study.

### 2.6 Clinical data collection

Maternal clinical data will be collected from all participants (Table 4). For sub analysis, participants in the study group can be assigned to one of three groups: (1) CAM, (2) FGR, and (3) no suspicion of CAM or FGR.

**Table 4:** Overview of maternal data collection.

Type of data	Specification
Standard demographic information	Maternal age, medical history, gravidity, parity, mode of delivery, duration of pregnancy
Maternal complications	Complications occurring during pregnancy or after delivery. E.g., detailed health report of suspected CAM and FGR.
Medication practices	Administration of medication before/during/after delivery, including oral/intravenous antibiotics, insulin, and anti-hypertensives. Vaccinations received during pregnancy.
Clinical data	Clinical signs of CAM, including maternal fever (>38 °C), tachycardia, abdominal pain, elevated white blood cell count and/or elevated CRP, blood culture results, fetal tachycardia or other signs of fetal distress in CTG.
Placenta pathology reports	Signs of maternal vascular malperfusion, fetal vascular malperfusion, acute or chronic CAM, and villitis of unknown etiology
Fetal ultrasound reports	HC, AC, EFW, FL, HC/AC, FL/HC, UADV
Microbial swabs	Taken before/after delivery. E.g., vaginal swab to assess maternal colonization with Group B *Streptococcus*.
Amniotic fluid collection	Detailed description of collection of AF, including mode of collection (e.g., in case of CS before or after amniotomy, rupturing of membranes prior to delivery, storage conditions).

AC, abdominal circumference; AF, amniotic fluid; CAM, chorioamnionitis; CS, cesarean section; CTG, cardiotocography; CRP, C-reactive protein; EFW, estimated fetal weight; FL, femur length; HC, head circumference; UADV, uterine artery Doppler velocimetry.

#### 2.6.1 Chorioamnionitis

Two separate definitions will be used to assess CAM: histopathologic CAM and clinical CAM. For histopathologic CAM, the diagnosis is based on histopathologic examination of the placenta (37). For clinical CAM, the diagnosis is based on the clinical signs (unexplained fever of 38 °C or more in combination with uterine tenderness and/or irritability, fetal and/or maternal tachycardia, and purulent or malodorous AF or vaginal discharge) as well as laboratory findings (e.g., leukocytosis), as defined by The American College of Obstetricians and Gynecologists (Table 5) (38).

**Table 5:** Definition of clinical chorioamnionitis, as defined by the American college of obstetricians and gynecologists (ACOG).

Criteria of clinical chorioamnionitis
Unexplained fever of at least 38 °C (rectal measurement)
*In combination with the following clinical indicators:* - Uterine tenderness and/or irritability - Fetal tachycardia (>160/min or increase of >30/min) - Maternal tachycardia (>100/min or increase >30/min) - Purulent or malodorous amniotic fluid or vaginal discharge - Leukocytosis (>15 × 10^6^/L)

#### 2.6.2 Fetal growth restriction

FGR is frequently defined as birth weight (BW) <10th percentile according to the Fenton growth charts (39). However, up to 70% of the small-for-gestational infants are constitutionally small but otherwise healthy fetuses. In contrast, some fetuses growing >10th percentile may still have growth restriction due to uteroplacental insufficiency. Therefore, the international consensus definition by Gordijn et al. (40) will be used for strict diagnosis of FGR (Table 6). For early growth-restriction (<32 weeks of gestation), the definition is as follows: abdominal circumference (AC) <3rd percentile and/or estimated fetal weight (EFW) <3rd percentile, *or* absent end-diastolic flow (AEDF) in the umbilical artery, *or* AC and/or EFW <10th percentile in combination with pulsatility index (PI) >95th percentile in either the umbilical artery or uterine artery (38, 40).

**Table 6:** International consensus definition of early fetal growth restriction (gestational age <32 weeks), as defined by Gordijn et al. (40).

Criteria of early fetal growth restriction (<32 weeks of gestation)
AC <3rd percentile and/or EFW <3rd percentile
*or* AEDF in the umbilical artery
*or* AC and/or EFW <10th percentile *in combination with* PI > 95th percentile in either the umbilical artery and/or uterine artery

AC, abdominal circumference, AEDF, absent end-diastolic flow, EFW, estimated fetal weight, PI, pulsatility index.

### 2.7 Neonatal data and sample collection

Neonatal clinical data and samples will be collected in line with the eMINDS study. In brief, neonatal data in the first 29 days of life will include GA, BW, surfactant administration, antibiotic and probiotic administration, feeding practices (e.g., time to full enteral feeding, feeding type, feeding volumes), and detailed description of relevant comorbidities (e.g., NEC and sepsis). NEC is defined by Bell's staging; only stage 2A or higher cases will be included (41). Sepsis is defined as isolation of bacterial and/or fungal pathogen from blood and/or cerebrospinal fluid, together with signs of generalized infection, and intention to threat with antimicrobials for 5 or more days (42). Early-onset sepsis is distinguished from late-onset sepsis, defined as occurring, respectively, within and after the first 72 h of life (42). Additionally, fecal samples will be collected and assessed, as specified in Table 1 (e.g., meconium/first stool sample, day 7, day 14, day 21, and day 28). Reasons and timing for missing data or partial follow-up fecal samples will be collected (e.g., mortality, comorbidities, transfer to another hospital). All available samples will be included in analyses (i.e., infants with partially longitudinally collected fecal samples will not be excluded *a priori*).

### 2.8 Sample storage and analysis

Following collection, each AF sample will be visually inspected for blood contamination and observations will be recorded. Hemoglobin levels will be measured in AF to quantify the severity of blood contamination, which is taken into account in downstream analyses. Additionally, low-speed centrifugation prior to long-term storage will aid in the removal of erythrocytes from AF (at 1,000 g for 15 min at room temperature, preferably before 4 h following AF collection). Both uncentrifuged sample, supernatant, and pellet will be stored at −80 °C. In the unlikely instance that AF cannot be immediately stored at −80 °C, AF will be stored at 4–8 °C prior to storage at −80 °C. AF will be transferred to −80 °C for long-term storage as soon as possible, preferably within 24 h of collection. The time between AF collection and −80 °C storage will be recorded. Characterization of AF will include at least microbial and metabolic profiling. The left-over of the sample will be refrozen at −80 °C and kept for future studies to increase our understanding of the impact of AF on the GI development.

### 2.9 Microbial and metabolic profiling

Characterization of AF will include at least microbial and metabolic profiling. Additional compositional analysis of AF may be indicated in the future focusing on other components (e.g., cytokines, growth factors, hormones). In brief, Intergenic Space profiling (IS-pro) assay, a PCR-based technique (inBiome, Amsterdam, the Netherlands), will be used for microbial profiling. Metabolic profiling will be conducted with Liquid Chromatography-Mass Spectrometry (LC-MS).

#### 2.9.1 Microbial profiling

IS-pro will be conducted in collaboration with inBiome (Amsterdam, the Netherlands). The IS-pro technique utilizes the length and sequence variations within the 16S-23S rDNA interspace region to identify bacterial species (43). IS-pro is a rapid technique with potential for clinical implementation. IS-pro is widely used to characterize (low-biomass) bacterial communities in physiologically sterile samples, such as pericardial fluid, synovial fluid, and blood (44–46). This technique has also been optimized for microbiota profiling of preterm fecal samples, demonstrating a high concordance between IS-pro and 16S rRNA sequencing as well as improved species-level resolution (47) The extensive IS-pro annotation database has been curated for sterile samples and covers common bacteria found in AF, such as *Escherichia coli* and Group B *Streptococcus*. For IS-fragments that cannot be annotated with the current database, additional high-resolution sequencing can be performed to refine and expand the database specifically for AF.

In short, ∼1–2 mL of AF is needed for IS-pro analysis. After bacterial DNA isolation, 10 µL of eluted DNA will be amplified using a PCR reaction. Using multiple phylum-specific fluorescently-labeled forward primers and unlabeled reverse primers, the IS-region is amplified and labelled per phylum. The forward primers, contained within the FIRBAC and PROTEO + IC mastermix 2.0 (inBiome), are targeted towards the Firmicutes, Actinobacteria, Fusobacteria and Verrucobacteria phylum group, the Bacteroidetes phylum, and the Proteobacteria phylum. 2.5 µL of each PCR product is prepared for fragment analysis through addition of 20 µL of eMix (inBiome). IS-fragments are separated and detected by capillary electrophoresis using ABI Prism 3130XL Genetic Analyzer (Applied Biosystems). The resulting data consists of peak profiles with different colors relating to the phyla and length signatures corresponding to specific bacterial species. The ABI Prism 3500XL data is analyzed and translated into quantified annotated bacterial identifications using Antoni Lab Cloud software, which denoises data and aligns the IS-fragments to taxa by means of a proprietary matching database (inBiome).

All samples will be handled using strict clean-lab procedures. A negative control will be included with each DNA extraction run to monitor potential contamination introduced during the microbial analysis process. For each batch of materials used for AF collection, we will randomly sample and process unused items as procedural controls to detect potential contamination introduced during the collection procedure. Additionally, all microbial AF profiles, considering the collection method, will be thoroughly assessed for potential contamination using a validated proprietary algorithm for low-biomass samples, which applies well-established bacterial abundance thresholds. Additionally, the samples will be screened for bacterial taxa commonly associated with skin or vaginal microbiota (e.g., *Staphylococcus* spp., *Gardnerella vaginalis*), as well as taxa known to be common laboratory contaminants.

#### 2.9.2 (Un)targeted metabolomics

LC-MS will be conducted in collaboration with the Department of Laboratory Medicine (Amsterdam UMC, location AMC, Amsterdam, the Netherlands). Notably, samples with substantial blood contamination will be excluded from metabolite analysis (e.g., samples containing >0.1 mmol/L hemoglobin), as the source of metabolites (AF vs. maternal blood) cannot be reliably determined in these instances. Depending on the technique, up to 2 mL AF is required for the analysis. Untargeted and/or targeted metabolomics will be conducted on the collected samples. Targeted metabolomics will include amino acid compositional analysis (7), with the possibility of extending the analysis to other targeted metabolites in the future. Amino acids are of interest because they are essential for fetal growth and gut development, serving as building blocks for protein synthesis and signaling molecules that may modulate immune responses (48–50). Their dynamics in AF, particularly in the context of preterm delivery and conditions such as CAM, remain poorly understood.

Amino acid analysis will be conducted using stable-isotope dilution LC-MS. Amino acids that will be measured include: alanine, citrulline, glutamate, glutamine, glycine, histidine, isoleucine, leucine, lysine, methionine, ornithine, phenylalanine, proline, serine, taurine, threonine, tryptophan, tyrosine, valine, and α-amino adipic acid. Amino acid concentrations will be quantified using commercial available calibrators together with the use of isotope-labelled internal standards. Amino acids will be measured using a Waters Acquity UPLC BEH C18 column (1.7 µm particle size) on a Vanquish UPLC system (Thermo Scientific) coupled to a TSQ Quantiva tandem mass spectrometer equipped with an electrospray ionisation. A 10 µL amino acid panel calibrator (Chromsystems) will serve as a quantitative reference. LC-MS data will be processed using Thermo Scientific Chromeleon software. To monitor and correct for batch-to-batch effects, a pooled AF sample will be created from several individual samples, aliquoted, and included in each measurement batch as a quality control to track instrument drift, technical variability, and facilitate normalization of metabolite intensities across batches.

### 2.10 Analysis plan

For bacterial analysis, relative abundances will be compared between the subgroups. Alpha-diversity will be calculated for each phylum and all phyla combined. Beta-diversity will be calculated using analysis of similarity based on Bray-Curtis distance. For both bacterial and metabolic data, Principle Coordinate Analysis will be used to assess the variation between samples and to visualize potential clusters by clinical features. Stratified analyses will be conducted taking into account collection method (e.g., samples collected via amniocentesis vs. at time of delivery, vaginal delivery vs. CS, CS through intact membranes vs. after rupturing of membranes). To provide further insight into the key identified metabolites and bacterial species, the components will be assessed in the reference group. We aim to determine the temporal behavior of the proposed key components in both healthy pregnancies (normal trajectories) as well as pregnancies complicated with FGR and CAM. Prior to analyses, data will be normalized according to the used technique. For bacterial profiling, relative or absolute abundances will be transformed as needed for specific subanalyses. For metabolite analysis, metabolite concentrations will be standardized across samples.

Secondarily, we will determine whether the groups of interest can be distinguished from each other using the principal components with (non-)parametric tests. To check for potential confounders (e.g., collection mode, GA, BW, feeding type, antibiotics), the association between potential confounders and outcomes of interest will be assessed. The association between the confounder and the compound of interest will be tested by Chi square test (for categorical variables) and ANOVA (for continuous variables). Logistic regression models will be used to adjust for confounding variables. To assess the AF composition in relation to the gut microbiota, associations will be tested by fitting generalized linear models of the microbiome feature on each component of interest, adjusting for confounders. Noteworthy, multivariate regression analyses require an adequate sample size and should be used cautiously due to the risk of overfitting and reduced statistical power, which can lead to biased findings. Multiple testing correction will be applied where appropriate to control type I error in multivariate or high-dimensional analyses; however, given the exploratory nature of the study, findings that do not remain significant after correction will still be reported for hypothesis generation.

### 2.11. Data handling and confidentiality

Data will be collected and processed in accordance with the General Data Protection Regulation (EU) 2016/679 and General Data Protection Regulation (GDPR). Every obstetric patient and their infant(s) participating in this study are assigned a unique study number. The key for description is kept at the center of birth. Only the main researchers are able to access this encrypted information. All pseudo-anonymized data is collected by the research team from the electronic patient files recorded into an electronic case report form on CASTOR EDC platform. Double data entry checks will be performed to ensure accuracy of the data. No later than 6 weeks after completion of all clinical data and sample analysis, the Castor EDC database will be locked. To ensure consistency between the AMFIBIE and eMINDS datasets, outcome definitions will be aligned wherever possible. The AMFIBIE study provides maternal and obstetric data, while the eMINDS study provides neonatal data. Shared variables between the datasets will be standardized using uniform definitions, measurement units (if applicable), and coding structures across both datasets to ensure consistency in analyses. Similarly, samples from both studies will be handled under comparable conditions (e.g., identical storage temperature and limited freeze–thaw cycles) and analyzed within the same time frame to minimize potential effects of storage duration and other temporal factors on measured outcomes.

## 3 Discussion

To our knowledge, the AMFIBIE study is the first to comprehensively characterize AF collected during extremely preterm deliveries, focusing on a subpopulation of infants exposed to CAM and/or FGR. Through microbial and metabolic characterization and through assessment of the relationship between AF profiles and the neonatal gut microbiota, we aim to elucidate the contribution of AF to the GI development and the gut microbiome.

We expect to identify distinct AF profiles, associated with extremely preterm birth. By employing a molecular microbial detection technique, we will likely detect bacterial species that may have been overlooked by traditional culturing. In parallel, metabolic profiling will provide valuable insights into the underlying pathophysiological processes. Compositional analysis may aid in the identification of components involved in the GI tract development, such as intestinal epithelial cell proliferation and the formation of the fetal intestinal barrier. Further *in vitro* studies will be required to establish causal relationships between these components and GI tract development as well as the pathogenesis of GI-related disorders. Additionally, we aim to link AF profiles to gut colonization patterns. In particular, infants exposed to CAM may exhibit distinct microbial signatures compared to unexposed preterm infants. Such alterations in early gut colonization may contribute to increased susceptibility to gut-related disorders in this population (2, 3). Microbiota alterations have been previously identified in infants with GI-associated disease (1–4). The current study aims to further explore whether similar patterns can be identified in preterm infants exposed to intra-uterine conditions such as CAM, and whether these findings may be linked to their AF profile.

Since our population consists of extremely preterm infants, sufficient AF may not always be available, such as after PPROM (∼30% to 40% of preterm deliveries) (32). The rapid pace required during extremely preterm deliveries may result in a lower-than-expected inclusion rate. Additionally, selection bias may be introduced, as AF collection is likely to be more successful during CS compared to vaginal deliveries, with CS rates among preterm births being as high as 37% (51). If this bias is indeed present in the final cohort, it represents a limitation that should be acknowledged and may restrict the generalizability of our findings to the broader population. Based on limited literature, it is not possible to determine in advance whether the planned sample sizes will provide sufficient samples for all planned subgroup analyses. The final number of samples per subgroup may be too small based on the current estimated sample size for the study group (*n* = 125). It may therefore be necessary to expand the sample size in future follow-up studies. Findings from the current exploratory study may guide more in depth hypothesis generation and the design of future larger-scale validation studies.

Notably, it may not always be feasible to collect AF samples without contamination by maternal blood. Blood contamination may complicate both microbial and metabolic analyses. Adequate preprocessing of such samples may be necessary to remove the erythrocytes prior to analysis. To mitigate these issues, intermediary analyses will be performed. Moreover, microbial contamination with urogenital microbiota during vaginal delivery or with skin microbiota during CS may complicate interpretation of our findings. In certain cases, it may be challenging to discern whether microorganisms detected in AF originate from the intra-amniotic environment or result from exogenous contamination introduced during delivery. To minimize this risk, the obstetric doctor will be instructed to collect the AF samples using only sterile materials. In the case of CS, sampling will be performed through intact membranes whenever possible to further reduce contamination. Nevertheless, contamination remains an inherent risk when AF is collected intrapartum, as opposed to via transabdominal amniocentesis, and cannot be completely eliminated. Therefore, the specific mode and circumstances of AF collection will be considered when interpreting the microbial profiles of the samples. These challenges may affect the robustness of statistical analyses and limit the ability to generalize our findings. Due to the clinical urgency inherent to preterm birth, the current study does not include sterile control samples or environmental monitoring, which could further strengthen contamination assessment in low-biomass studies. Future studies building on this work may incorporate such controls to improve the robustness and interpretability of the findings. However, we argue that due to the exploratory nature, our findings will still provide valuable preliminary insights that can guide future research on this topic.

Through addressing the challenges and opportunities associated with AF collection during extremely preterm delivery as well as the subsequent microbial and metabolic analysis, we aim to advance research on the role of AF in early-life development. We expect to offer novel insights into the impact of intestinal exposure of AF on the GI development, the pathogenesis of neonatal GI-related disease, and neonatal intestinal colonization. These findings may guide future studies to discover perinatal diagnostic biomarkers identifying preterm infants at increased risk for GI-related diseases. More in-depth characterization, combined with *in vitro* studies exploring the impact of AF on the gut barrier and disease development, may expand upon this research project.

## 4 Ethics and dissimination

**Figure 2:**
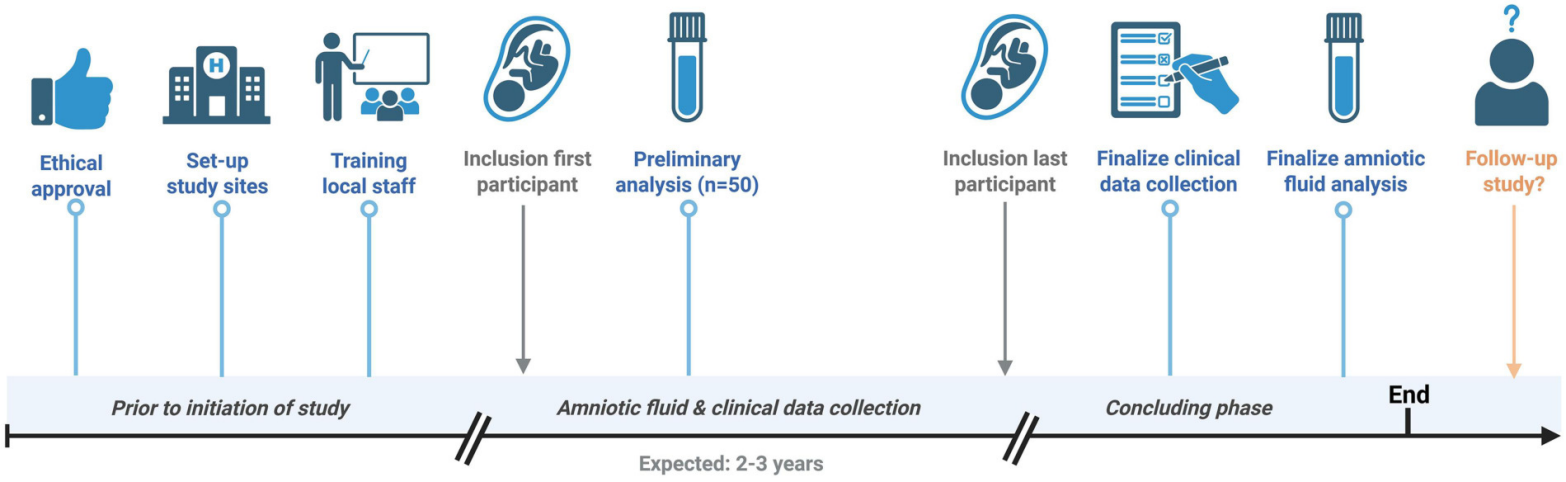
Recruitment and analysis milestones. Created with Biorender.com.

The current study was approved by the METC of the Máxima Medical Center in Veldhoven, the Netherlands, on September 26th 2024 (W24.042). The study was registered at the Central Committee on Research Involving Human Subjects (CCMO) in the Netherlands (NL86579.0152). Additionally, the study was registered in a public clinical trial registry (NCT07152106). Recruitment of patients began in October 2024 at the Máxima Medical Center and in January 2025 at the Amsterdam UMC. The first results are anticipated by October 2026. Figure 2 displays the recruitment and analysis milestones. Study findings will be disseminated through peer-reviewed journal articles as well as national and international conference presentations.
